# User-centered Design and Formative Evaluation of a Web Application to Collect and Visualize Real-time Clinician Well-being Levels

**DOI:** 10.1055/s-0044-1779698

**Published:** 2024-02-21

**Authors:** Derek Shu, Catherine T. Xu, Somya Pandey, Virginia Walls, Kristen Tenney, Abby Georgilis, Lisa Melink, Danny T.Y. Wu, Jennifer Rose Molano

**Affiliations:** 1 Department of Biomedical Informatics, College of Medicine, University of Cincinnati, Cincinnati, Ohio, United States; 2 Medical Doctorate Program, College of Medicine, University of Cincinnati, Cincinnati, Ohio, United States; 3 Medical Sciences Baccalaureate Program, College of Medicine, University of Cincinnati, Cincinnati, Ohio, United States; 4 University of Cincinnati Health, Cincinnati, Ohio, United States; 5 University of Cincinnati Blush Ash College, Cincinnati, Ohio, United States; 6 Department of Neurology and Rehabilitation Medicine, College of Medicine, University of Cincinnati, Cincinnati, Ohio, United States

**Keywords:** care teams, interfaces and usability, data visualization, other clinical informatics applications

## Abstract

**Background** Clinician burnout is increasingly prevalent in the health care workplace. Hospital leadership needs an informatics tool to measure clinicians' well-being levels and provide empirical evidence to improve their work environment.

**Objectives** This study aimed to (1) design and implement a web-based application to collect and visualize clinicians' well-being levels and (2) conduct formative usability evaluation.

**Methods** Clinician and staff well-being champions guided the development of the Well-being Check application. User-centered design and Agile principles were used for incremental development of the app. The app included a customizable survey and an interactive visualization. The survey consisted of six standard, two optional, and three additional questions. The interactive visualization included various charts and word clouds with filters for drill-down analysis. The evaluation was done primarily with the rehabilitation (REHAB) team using data-centered approaches through historical survey data and qualitative coding of the free-text explanations and user-centered approaches through the System Usability Scale (SUS).

**Results** The evaluation showed that the app appropriately accommodated historical survey data from the REHAB team, enabling the comparison between self-assessed and perceived team well-being levels, and summarized key drivers based on the qualitative coding of the free-text explanations. Responses from the 23 REHAB team members showed an above-average score (SUS: 80.22), indicating high usability of the app.

**Conclusion** The Well-being Check app was developed in a user-centered manner and evaluated to demonstrate its effectiveness and usability. Future work includes iterative refinement of the app and designing a pre-poststudy using the app to measure the change in clinicians' well-being levels for quality improvement intervention.

## Introduction

Well-being is multidimensional and is influenced by physical, emotional, mental, social, and spiritual factors that interact together within one's environment and circumstances. 1 Many workplace factors, such as workplace culture, connection to a greater purpose, and efficiency, contribute to clinician well-being and are also involved in influencing clinician burnout prevalence. 2 3 Burnout has been defined as a “work-related syndrome” commonly characterized by high levels of emotional exhaustion, depersonalization, and dissatisfaction with one's career. 4 5 According to a 2020 physician burnout study, 64% of respondents reported that the COVID-19 pandemic had worsened their feelings of burnout. 6 This increase in burnout prevalence is not limited to physicians. A 2020 national survey of more than 20,000 health care workers found that 49% of respondents had burnout and 43% suffered from work overload. 7 Clinician burnout affects medical errors and field attrition, compromising the quality of patient care, and increasing costs related to medical errors, with ramifications emanating throughout the entire medical system. 8 9 Therefore, there is a pressing need to develop tools for health care administration to support clinicians in their workplace efficiently.

Risk factors for clinician burnout have been increasingly studied and documented in recent years, with work–life conflict, lack of autonomy, increased work hours, and toxic workplace culture being the most studied factors. 10 11 12 Previous studies have developed and investigated interventions (e.g., wellness programs) to reduce clinician burnout. For example, Aggarwal et al incorporated a low-cost yet effective wellness program into the residency training curriculum and found a decrease in severity and prevalence of resident burnout while increasing resilience and happiness. 13 On the other hand, the pervasive use of health information technology (HIT) over the last decade throughout the health care systems has become part of everyday care. HIT collects, stores, retrieves, and manages clinical data and other health-related information. Although HIT, such as electronic health records, can contribute to clinician burnout by increasing clinicians' workload and work hours, HIT can also play a role in measuring burnout and be used as a target for interventions to reduce burnout. 14

One way of utilizing HIT to reduce burnout is through data visualizations, which can provide easy-to-understand measurement of burnout and aid in identifying the best targets for interventions. That is, through HIT, well-being data in a clinical work environment can be visualized in a dashboard to show insights and inform improvements. Current data visualization modalities for well-being incorporate structured data from wearable technology or other sensors and focus on the general population. 15 16 However, a gap remains in developing tools and dashboards in a user-centered manner and enabling real-time collection and feedback of clinicians' well-being levels. Without such tools and real-time feedback, clinicians would neither be aware of their burnout due to medical workplace culture nor would they have the empirical evidence to act upon it using personalized interventions. 17

To address this gap, this study aimed to design and develop an interactive, web-based application to collect and visualize the well-being levels of clinicians using both structured and unstructured data. This is a novel application, as, to our knowledge, there have not been any data visualization tools aiming toward helping clinicians understand their well-being levels at the individual and the team level. Moreover, adding the analysis of unstructured data in conjunction with structured data will help generate a new understanding of key drivers that affect clinicians' well-being.

## Methods

### Clinical Setting

This study was conducted in a leading adult academic health center (AHC) with more than 700 beds in the midwestern United States. The AHC introduced the Epic EHR system in 2012 and had two major locations with over 15,000 employees. The AHC has employee well-being services (EWS) and maintains a strong partnership with the College of Medicine at the university through care delivery, clinical research, and medical education. The College of Medicine and the AHC have formed well-being advisory councils for faculty members and resident trainees, respectively. These advisory councils have been collecting well-being data periodically through online surveys.

### Study Design

**Fig. 1:**
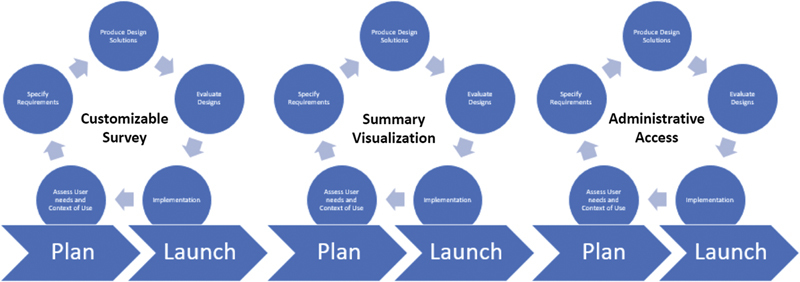
Application development strategy using user-centered design and Agile software development.

As shown in Fig. 1 , the methodology was conducted in the three major steps. While conducting the study, Agile software development principles were combined with user-centered design methodology to prioritize creating an application that is adaptable to an individual user's needs, keeping ease of usability at the forefront. Agile software development also allows for flexibility in the development process through incremental testing. These principles were utilized to prioritize the needs of clinicians and administrators while efficiently developing and evaluating the usability of the application through multiple rounds of testing.

### Application Development

The Well-being Check application (the app, hereafter) was developed utilizing user-centered design 18 and Agile software development principles (e.g., incremental/interactive development and continuous testing) 19 with a responsive design for mobile devices. The application was created using Python Flask and hosted Microsoft Azure.

**Fig. 2:**
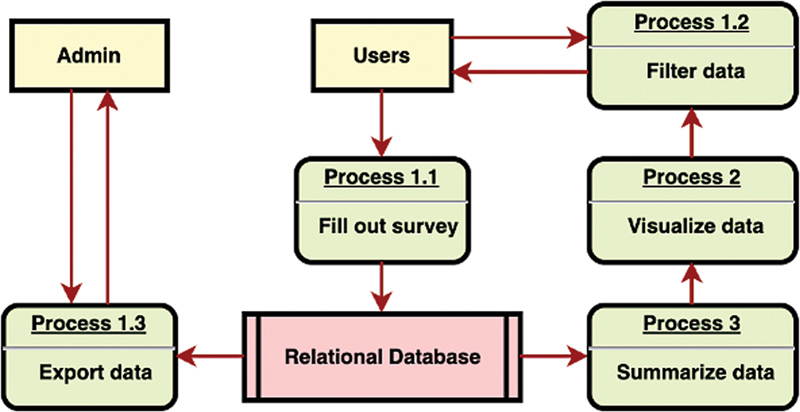
Data flow diagram of the Well-being Check App.

**Fig. 3:**
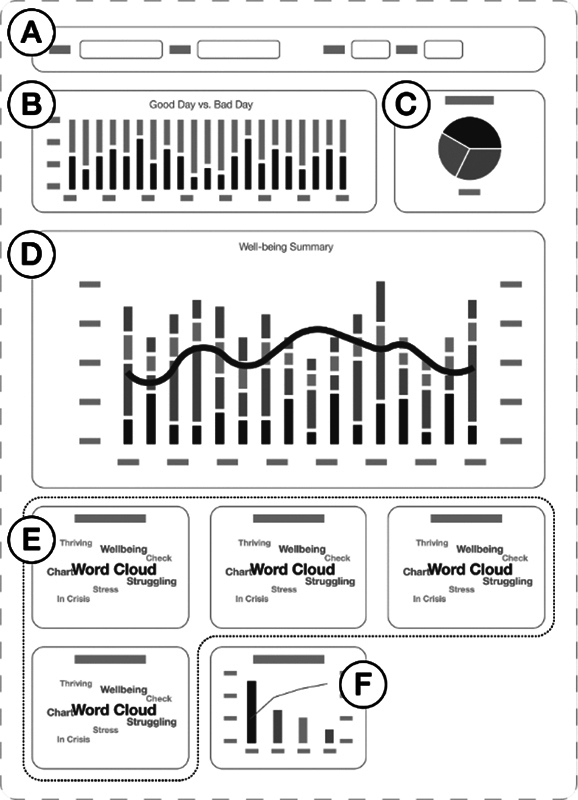
Mock-up of the interactive visualization in Well-being Check App. The presentation incorporated filters based on user-input data, enabling detailed analysis according to team roles and dates ( **A** ). It also featured charts that captured trends in well-being over time ( **B** – **D** ), word clouds illustrating the frequency of words in free-text explanations ( **E** ), and highlighted the key drivers ( **F** ).

The app was developed in three cycles, namely Customizable Survey, Summary Visualization, and Administrative Access ( Fig. 2 ). The combination of user-centered design and Agile software development principles can address user needs and ensure ease of use while delivering the product in an incremental fashion. Moreover, the app was designed to provide easy access to a customizable survey with an interactive visualization summarizing the well-being levels in real-time. Fig. 3 shows the data flow of the app. Prior to the development, a core team was formed including a physician champion, a medical informatician, and several AHC EWS and patient experience experts. This team met weekly to discuss the strategic plan for collecting and presenting the well-being data while reviewing the app prototypes and making recommendations. The app was developed and refined iteratively between September 2021 and February 2022 (6 months) and piloted with three clinical teams: intensive care unit (ICU) nurses, nurse leaders (LDR), and a rehabilitation (REHAB) team. The major components of the app (Customizable Survey and Interactive Visualization) are described in the following subsections.

#### Customizable Survey

The survey was designed with standardized and customizable questions to maintain cross-team comparison while fulfilling individual team needs ( Table 1 ). 20 The survey consisted of three sections: (1) Standardized multiple choice questions with optional free response fields for elaboration, which were guided by the best practices of the Stress First Aid's Stress Continuum Model and the Institute for Healthcare Improvement's Joy in Work, (2) Optional shift and role-related questions, and (3) Additional multiple choice or free-text questions. Regular meetings were conducted with each clinician team or its representative (noted below) to determine the type and description of the questions that would best address their needs. Moreover, the survey was designed to be routinely administered and to collect data anonymously. The survey was made available to team members either through daily email notifications or Quick Response codes posted in the work areas.

**Table 1:** Survey question types and examples from the Well-being Check App

Type	Team(s) using	Question category	Questions
Standard	All	Multiple choice a	1. Are you having a good day?
		Multiple choice a	2a. What is your stress level today? c
		Free text	2b. Please briefly explain your stress level.
		Multiple choice a	3a. What do you think the team's stress level is today? c
		Free text	3b. Please briefly explain the team's stress level.
		Free Text	4. Please offer any suggestions for improvement.
Optional	REHAB, ICU	Multiple choice b	What is your role in this team?
		Multiple choice b	What shift did you work today?
Additional	REHAB	Free text	What was your win of the week and why?
	LDR	Multiple choice	How often do you lose sleep over work-related issues?
		Multiple choice	How often do you feel anxious during the day?

Abbreviations: ICU, intensive care unit; LDR, leader; REHAB, rehabilitation.

a Required.

b Required, if exists.

c Survey participants were given four answers to choose from, with their responses being mapped to the Stress Continuum Model (Green, Yellow, Orange, and Red). 24 Answer choices were consistent in wording between all the marked questions.

#### Interactive Visualization

The visualization design followed Munzner's nested model, 21 which includes (1) domain problem characterization, (2) the data abstraction design, (3) encoding/interaction technique design, and (4) algorithm design. The problems and use cases of the interactive visualization were discussed and refined with the core team, and then with the three clinical teams. Following this, structured survey data were summarized statistically while the unstructured survey data (free text) were processed using the Python Natural Language Toolkit to generate tokens and then word clouds to identify common themes within responses. Next, the encoding and interactivity were planned by the designer of the corresponding author's lab based on the group discussion with the core and clinical team. As shown in the mock-up ( Fig. 3 ), the visualization included user input data filters to allow for drill-down analysis by team roles and dates ( Fig. 3A ), charts summarizing well-being trends over time ( Fig. 3B–D ), word clouds representing the word frequencies of free-text explanations ( Fig. 3E ), and the key drivers ( Fig. 3F ).

**Fig. 4:**
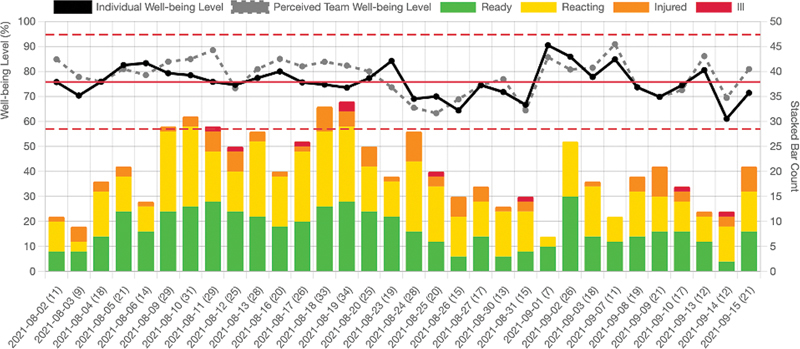
Summary visualization for the REHAB well-being level. REHAB, rehabilitation. The interactive visualization included filters utilizing user-input data, facilitating in-depth analysis by team roles and dates. It showcased charts that depicted well-being trends over time, word clouds visualizing word frequencies in free-text explanations, and emphasized the primary drivers.

Lastly, an algorithm was developed to convert categorical well-being levels into numerical values by well-being category (Green: Thriving = 3 points; Yellow: Surviving = 2 points; Orange: Struggling = 1 point; Red: In Crisis = 0 point) and sum them per day. This number was then divided by the maximum well-being score that assumed that everyone surveyed on that day was thriving (Thriving = 3 points) to generate the well-being level of that date. All well-being levels in the observation period were then plotted as a line chart ( Fig. 3D ) and laid on top of a stacked bar chart based on the counts of the original well-being categories. It is worth noting that two trend lines of well-being levels were plotted. The first was drawn in a solid line and aggregated from the “self-assessed” well-being levels (Question 2a in Table 1 ). The other was drawn in a dotted line and aggregated from the “perceived” team well-being levels (Question 3a in Table 1 ).

### Formative Evaluation

The app was piloted with three clinical teams (ICU, LDR, and REHAB) between January and June 2022. Separate apps were created and personalized to each team's needs, and team members were requested and reminded to submit one survey response daily. App changes (adding/changing survey questions, adding/removing visualization figures, etc.) were made in response to clinician team requests. While the ICU and the LDR teams provided their feedback to the group, their interest in using the app decreased over time due to the heavy clinical workload and other priorities. This decision made the research team cautious about the potential burden that our app may add to the participating clinicians. As a result, the formative evaluation of the app was mainly conducted with the REHAB team, which included three groups of clinicians (Physical Therapist or PT, Occupational Therapist or OT, and Speech Therapist or ST). The formative evaluation consisted of data-centered and user-centered approaches as described in the following two subsections. The data-centered evaluation was conducted in the first and second development cycles while the user-centered evaluation (System Usability Scale [SUS]) was conducted in the second development cycle ( Fig. 1 ). The evaluation of the third cycle was skipped since administrative access was only granted to the research team.

#### Data-centered Evaluation

The goal of the data-centered evaluation was to (1) ensure that the app could accommodate historical survey data and generate interactive visualization as intended and (2) to show qualitative coding results based on the free-text explanations. The survey data were collected using a Research Electronic Data Capture (REDCap) form between July 26, 2021 and September 17, 2021 (8 weeks). The historical survey data only had a subset of questions compared to the Well-being Check app, which included four standard questions (Questions 2a, 2b, 3a, and 3b in Table 1 ) and one optional question (clinical role: PT/OT/ST). Since the survey collected the self-assessed and the perceived team well-being level (Questions 2a and 3a), the numeric discrepancy between the two well-being levels was quantified by the Mean Absolute Error (MAE), which calculated the absolute difference of the two well-being scores per day and took an average of these numbers.

Qualitative coding was conducted manually on the free-text explanation of the self-reported well-being levels using the taxonomy (key drivers) developed by the Institute for Healthcare Improvement (IHI). The key drivers include the following nine IHI categories with an additional nonwork-related miscellaneous category: (1) Camaraderie and Teamwork, (2) Choice and Autonomy, (3) Daily Improvement, (4) Meaning and Purpose, (5) Participative Management, (6) Physical and Physiological Safety, (7) Real-time Measurement, (8) Recognition and Rewards, (9) Wellness and Resilience, (10) Nonwork-related. 22 The coding was focused only on the textual comments of the lowest self-assessed well-being levels (Surviving and In Crisis). The coding and discrepancy resolution were done by the core team members in a group discussion.

#### User-centered Evaluation

The user-centered evaluation assessed the usability of the app through the SUS 23 with the REHAB team. The SUS was utilized for assessment to minimize the time required for participating clinicians to provide their insight given their constrained availability. This allowed informational data for refinement to be easily collected. The SUS survey was disseminated to all 52 REHAB team members, including 21 PTs, 20 OTs, and 11 STs. The SUS survey contained 10 questions, each scored from 1 to 5 (from Strongly Disagree to Strongly Agree). The SUS composite score of each participant was calculated based on the definition, 23 with the highest possible score being 100. A system or application with an SUS score above 68 is deemed to have above-average usability. Furthermore, the SUS scores were grouped by clinical role (PT/OT/ST), summarized using a box plot, and compared statistically. If the distribution of the SUS scores was normal (Shapiro–Wilk's test), the mean SUS scores of the three groups were compared using pairwise *t* -tests with Bonferroni's correction. Otherwise, the median SUS scores of the three groups were compared using the nonparametric alternative (Mann–Whitney test). In addition, a group discussion with the team leaders was held to collect additional user feedback after distributing the survey.

## Results

### Data-centered Interactive Visualization

Fig. 4 shows the interactive visualization with filters for the REHAB team based on the historical survey data. The visualization successfully generated a stocked bar chart using the predefined color coding and a line chart representing the self-assessed and the team well-being levels during the time frame (the dotted and solid lines, respectively). The interactive visualization contained a set of filters (date, role, count, and workdays) to provide drill-down analysis.

### Data-centered Evaluation

Table 2 shows a summary of the self-assessed versus the perceived well-being levels. The STs had the highest MAE (10.89), followed by the PTs (7.44) and the OTs (6.68), meaning that the STs had a greater difference between perceived team and personal self-assessed well-being levels compared to PTs and OTs. Table 3 summarizes the qualitative coding based on the free-text explanations of the lower well-being levels ( *N* = 80). The overall response rate of the free-text explanations was 11.49% (80/696). Choice and Autonomy were the most frequently coded reason (46.25%), followed by Nonwork-related Causes (25.0%), and Wellness and Resilience (15.0%) when the well-being level was characterized as poor (Surviving or In Crisis). The overall response rate of the free-text explanations was 11.49% (80/696).

**Table 2:** Statistical summary of the discrepancy of the self-assessed versus the perceived team well-being levels in the rehabilitation team

Role	MAE	Proportion of self-assessed < Perceived team well-being level	Proportion of self-assessed > Perceived team well-being level
PT	7.44	13 (30.2%)	11 (42.3%)
OT	6.68	7 (16.3%)	13 (50%)
ST	10.89	23 (53.5%)	2 (7.7%)
All	8.32	43 (100%)	26 (100%)

Abbreviations: MAE, mean absolute error; OT, occupational therapist; PT, physical therapist; REHAB, rehabilitation; ST, speech therapist.

**Table 3:** Coding of the free-text explanation of self-wellbeing levels ( *N* = 80)

Code	Count (%)	Quote
Choice and Autonomy	37 (46.25)	“Low caseloads, multiple refusals which makes it difficult to meet productivity”
Nonwork-related	20 (25.00)	“Home life stress, children”
Wellness and Resilience	12 (15.00)	“Very tired, feeling burned out”
Physical and Psychological Safety	8 (10.00)	“Working with patients that are covid + . Very stressful. Worried I'm putting myself at risk and my family”
Participative Management	2 (2.50)	“Feel unsupported by management. Feel as if we are disposable and they don't care about me as a person”
Camaraderie and Teamwork	1 (1.25)	“MDs failing to acknowledge my presence or apologize for interruptions even if they consume 10+ minutes of my session”

### User-centered Evaluation

**Fig. 5:**
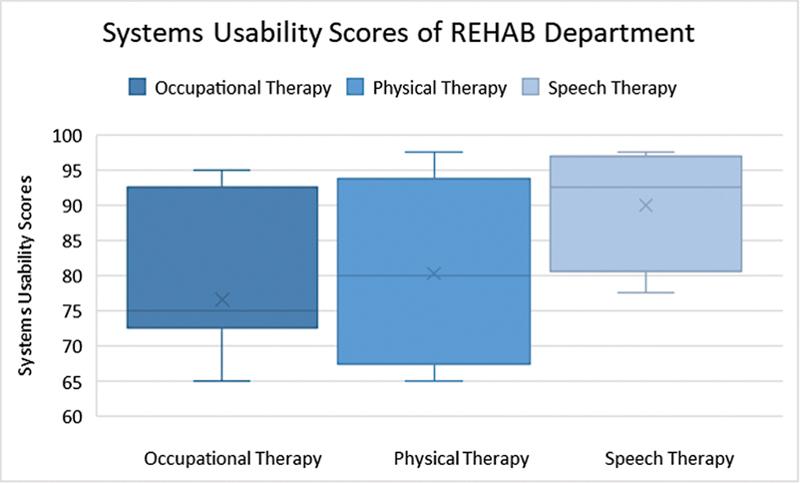
Distribution of SUS scores by clinical roles. SUS, System Usability Score.

The SUS survey collected 23 responses, including 8 PTs, 11 OTs, and 4 STs response rate (44.2%). The mean, median, and standard deviation of the overall SUS scores were 80.22, 82.50, and 15.59, respectively, indicating that the app's survey had above-average usability (>68). Further comparison of the SUS scores among the subteams (PT/OT/ST) showed no statistical significance between the groups while the PTs had a wider range of usability scores ( Fig. 5 ). In addition to the SUS scores, the discussion with the team leaders collected two pieces of feedback. First, the word clouds were not very informative and some of them were even confusing. Second, the wording of the well-being levels (Thriving, Surviving, Struggling, and In Crisis) may be suboptimal.

## Discussion

### Key Findings

In this study, we successfully developed an effective and easy-to-use, web-based application to collect and analyze the well-being levels of a clinical team in real-time. Through the user-centered design and Agile software development principles, three clinical teams helped mold and improve the customized survey and the interactive visualization of the app. Within our study, the REHAB engaged in the activities the most and provided their historical data as well as user feedback to help gauge the usability of the app. The data-centered and user-centered evaluation showed promising results. Discrepancies between the self-assessed and the perceived team well-being level were found in each subgroup of the REHAB team. The larger the MAE was, the more likely the team members had a wrong impression of the team's well-being level. How these discrepancies contribute to the work environment requires further study. With regards to the key drivers, Choice and Autonomy, Nonwork-related Causes, and Wellness and Resilience made up more than 85% of the explanations for REHAB team members who had poor well-being levels. In addition, the response rate to the free-text explanations indicated that participants were less likely to elaborate on open-ended questions. In future studies, guided questions may be implemented to gain a greater insight into well-being levels alongside generalized questions that allow for participants' candor. The team leaders suggested hiding the word clouds before figuring out a better way to deliver the information. Using the original category names of the Stress Continuum Model (Ready, Reacting, Injured, Ill) may better and more accurately describe the well-being levels. Meanwhile, the REHAB team leads shared concerns about the word clouds and the wording of the well-being levels. These findings highlight the opportunities for further investigations and can inform future interventions to improve well-being levels.

### Implications

The Well-being Check app can measure clinicians' well-being levels in real-time and provide the users with information that can prompt earlier interventions after the initial indicators of burnout are presented. With the application's capability of adapting its survey and visualization to address team structures and needs, our study creates an avenue that uses HIT to study and intervene on the well-being of all types of clinicians. Since the app can be used in any clinical team, it addresses the gap where previous studies on burnout and HIT have focused mostly on physicians without considering other types of health care providers. 14

The formative evaluation with the REHAB team elucidated many study-driven implications. Higher MAE scores represent an incongruence between the self-assessed and the perceived well-being levels, which may require further exploration of root causes contributing to these results, such as different priorities, situational factors, and systemic issues. 25 26 Through further investigation of this measurement, team leaders, with expertise in management and problem-solving, can identify isolated or persistent issues and stage small- or large-scale interventions to address team dynamics. Moreover, by categorizing free-text explanations of personal well-being levels, systemic themes can arise over time that can help diagnose problems with overall workplace culture. Through further investigation of this measurement, team leaders, with expertise in management and problem-solving, can identify isolated or persistent issues and stage small- or large-scale interventions to address team dynamics. Moreover, by categorizing free-text explanations of personal well-being levels, systemic themes can arise over time that can help diagnose problems with overall workplace culture. For example, if the category “Choice and Autonomy” characterizes most free-text explanations when well-being levels are low, team members may not feel empowered or supported to use their skills to independently practice in the clinical environment. 27 Teamwork interventions such as participation in the change process and perception changes may contribute to the increase in employee autonomy. Finally, the app had above-average usability as determined by the REHAB team. Therefore, using the app may not add extra burden to the clinicians.

### Limitations

Our study was limited to a single, large adult academic institution. More studies with different clinical teams and across health care systems should be conducted to determine the generalizability of our findings. Despite the absence of formal measurements to assess the validity or reliability of the survey questions, the development of the standard questions was informed by the best practices of the Stress First Aid's Stress Continuum Model and the IHI's Joy in Work. Additionally, the survey underwent a collaborative iterative refinement process facilitated by our well-being team, which consisted of several stakeholders to ensure that the questions were able to capture the well-being levels of the clinicians accurately. The qualitative coding of the free-text feedback enabled users to map the feedback to the drivers of clinician burnout. Our primary objective in the current study was to share the app design and to pilot the evaluation process. Regarding user-centered evaluation, the present study only used SUS because semistructured interviews, formative usability testing, participatory design, focus groups, and cognitive walkthrough require a significant amount of time from the participants. The study participants are clinicians who may already be experiencing a high level of burnout in the workplace and often have busy schedules. SUS was the most efficient and validated tool to gain insight into the usability of the application. However, this can be overutilized and not offer an in-depth understanding of the usability of a system alone. In the same vein, there were no qualitative interviews conducted with the clinical teams to gauge their interest and elicit thoughtful feedback about this app. Even though team members were given the chance to indicate the app's usability and provide textual feedback via the SUS, formal interviews could help elaborate on more nuanced feedback. The repetition of completing the survey daily may add an extra burden on the participating clinicians. Future completion of the surveys will consider this as a factor and create a plan to mitigate it. Finally, within our REHAB team, there were only four STs who participated in this study, creating potential skews or biases in the survey data.

### Future Work

In the future, both formative and summative usability testing will be conducted using other methods such as think-aloud, semistructured interviews, and eye-tracking. In addition, heuristic evaluation could examine whether a system follows known usability principles. We also hope to deploy this app with more clinical teams to improve its generalizability and start to demonstrate its effectiveness. As more clinical teams trial this app, different app adaptations such as survey questions, summary statistics, and visualization will be generated based on individualized needs and team structures. With the support of the interactive visualization, we anticipate that team leaders will be able to utilize the app to generate guidance and nurture their teams. Meanwhile, the app's integration into a health system's quality improvement effort and EWS infrastructure may help gather resources, enable the early detection of burnout or stress, and allow team leadership to intervene timelier and appropriately. Finally, we envision this app to be a critical tool in improving clinicians' well-being. Once systemic weaknesses have been identified, the four-step IHI framework for improving joy in work can be implemented to identify the root cause. 21 A pre- and postintervention study can then be conducted with the app to provide empirical evidence of observable and objective improvements.

## Conclusion

We developed a novel application with a customizable survey and an interactive visualization to collect and analyze clinician well-being levels in real-time. While the formative evaluation has demonstrated the effectiveness and usability of the application, it also identified several areas to intervene and explore, including the discrepancy between the self-assessed and perceived team well-being levels as well as interventions to address barriers to Choice and Autonomy. We will continue refining the Well-being Check app and use it as part of our methodology to assess and improve clinician well-being.

## Clinical Relevance Statement

Clinician well-being and burnout are increasingly studied entities in the medical community, intersecting with workplace culture, HITs, and patient care. The findings of the present study highlight the creation of a novel web application consisting of a customized survey and an interactive visualization that can collect and display clinician well-being levels in real-time. Pilot implementation of this application with a hospital rehabilitation team has helped to elucidate individual instances and team-specific causes of burnout. The app has the potential to be used by various clinical teams to help improve their well-being levels.
